# SIRT1 activation promotes bone repair by enhancing the coupling of type H vessel formation and osteogenesis

**DOI:** 10.1111/cpr.13596

**Published:** 2024-01-11

**Authors:** Zhikai Liu, Hanghang Liu, Shibo Liu, Bolun Li, Yao Liu, En Luo

**Affiliations:** ^1^ State Key Laboratory of Oral Diseases & National Center for Stomatology & National Clinical Research Center for Oral Diseases, West China Hospital of Stomatology Sichuan University Chengdu Sichuan China

## Abstract

Bone repair is intricately correlated with vascular regeneration, especially of type H vessels. Sirtuin 1 (SIRT1) expression is closely associated with endothelial function and vascular regeneration; however, the role of SIRT1 in enhancing the coupling of type H vessel formation with osteogenesis to promote bone repair needs to be investigated. A co‐culture system combining human umbilical vein endothelial cells and osteoblasts was constructed, and a SIRT1 agonist was used to evaluate the effects of SIRT1 activity. The angiogenic and osteogenic capacities of the co‐culture system were examined using short interfering RNA. Mouse models with bone defects in the femur or mandible were established to explore changes in type H vessel formation and bone repair following modulated SIRT1 activity. SIRT1 activation augmented the angiogenic and osteogenic capacities of the co‐culture system by activating the PI3K/AKT/FOXO1 signalling pathway and did not significantly regulate osteoblast differentiation. Inhibition of the PI3K/AKT/FOXO1 pathway attenuated SIRT1‐mediated effects. The SIRT1 activity in bone defects was positively correlated with the formation of type H vessels and bone repair in vivo, whereas SIRT1 inhibition substantially weakened vascular and bone formation. Thus, SIRT1 is crucial to the coupling of type H vessels with osteogenesis during bone repair.

## Graphical abstract

SIRT1 activation enhances the coupling of type H vessel formation with osteogenesis, promoting bone repair through the PI3K/AKT/FOXO1 signalling pathway. Inhibition of SIRT1 weakens vascular and bone formation, highlighting the crucial role of SIRT1 in the coupling process.

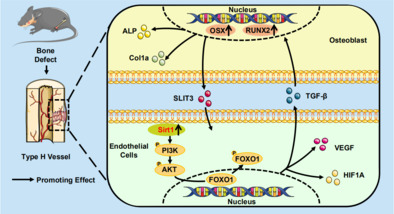

## 1 BACKGROUND

Bone defects can arise from infection, tumour resection, or traumatic injury, potentially having serious consequences, including fractures and deformities.^1^, ^2^ Extensive segmental defects compromise the normal biomechanical function of the bone structure, threatening the structural stability of the bone.^3^ Currently, autologous bone grafting, distraction osteogenesis, and exogenous regeneration are the most commonly used treatments for bone defects. However, these methods can fail to achieve satisfactory healing due, in part, to insufficient vascular regeneration.^4^, ^5^

Recent research has identified type H vessels as specific capillaries with high cluster of differentiation 31 (CD31) and endomucin (EMCN) expression.^6^ By coupling angiogenesis with osteogenesis, this capillary subtype maintains bone homeostasis. Moreover, the absence of type H vessels inhibits osteogenesis in regenerating tissues.^7^, ^8^, ^9^ Osteogenesis is regulated by the proliferation and differentiation of osteoprogenitors in type H vessels.^10^ Osterix (OSX) and runt‐related transcription factor 2 (RUNX2) are expressed by these osteoprogenitor cells.^6^, ^11^ RUNX2 plays a crucial role in the osseointegration process of dental implants with different surface treatments in periodontal stem cells, indicating its indispensable role in the osteogenesis process.^12^ Furthermore, high secretion of vascular endothelial growth factor (VEGF) by hypertrophic chondrocytes promotes new blood vessel formation.^13^ VEGF, as one of the most common angiogenic factors, is closely associated with cell adhesion, angiogenesis, and subsequent osseointegration processes of biomaterial implants.^14^ Osteogenesis and angiogenesis are intricately coupled through the interplay between Notch signalling and VEGF. However, it remains unclear whether regulatory factors enhance the coupling function between type H vessels and osteogenesis to further promote bone repair.

Sirtuin 1 (SIRT1) is an essential NAD(+)‐dependent deacetylase^15^ that regulates endothelial function and maintains endothelial homeostasis.^16^, ^17^ Furthermore, SIRT1 controls the angiogenic activity of endothelial cells (ECs) during angiogenesis. EC‐specific knockout of SIRT1 results in severe impairment of postnatal neovascularization in mice, confirming that the loss of SIRT1 inhibits EC sprouting and branching morphogenesis, slowing ischemia‐induced neovascularization.^18^ Additionally, Zhang et al. reported that the sustained release of SIRT1 activators restores the imbalanced bone homeostasis by regulating osteoblast/osteoclast differentiation.^19^ However, whether SIRT1 regulates type H vessels in bone defects remains to be elucidated.

Extensive research has been conducted using various co‐culture systems, primarily focusing on direct and indirect co‐culture systems. The direct co‐culture system involves the seeding of two different cell types in the same plate to create a 2D monolayer system. In contrast, the indirect co‐culture method utilizes semi‐permeable membranes like Transwell to seed cells at different levels, facilitating the exchange of extracellular matrix derivatives and signal transmission within the system. Regardless of the chosen method, optimizing the cell‐seeding density and ratio has consistently proved challenging. Nevertheless, Shah et al. utilized a single‐layer co‐culture system and found that a 5:1 co‐culture ratio between human umbilical vein endothelial cells (HUVECs) and osteoblasts positively impacts angiogenesis, whereas a 1:5 ratio results in higher mineralization rates.^20^ In contrast, De Moor et al.^21^ reported that when HUVECs are combined with fibroblasts or adipose tissue‐derived mesenchymal stem cells (MSCs) at a 1:9 cell ratio, viable and stable spheres are formed. However, spheres with a higher proportion of HUVECs tend to aggregate and exhibit poorer morphology. This is consistent with the findings of Ma et al.,^22^ suggesting that co‐culturing HUVECs with a higher proportion of MSCs prevents sphere formation. This study also found that a 1:1 co‐culture ratio of HUVECs to MSCs is optimal for achieving osteogenic and angiogenic differentiation. However, these studies lack relevant research on the co‐culture of primary osteoblasts and HUVECs. Moreover, the factors governing the angiogenic and osteogenic capabilities of this particular co‐culture system remain unclear.

In the current study, we hypothesized that SIRT1 plays a regulatory role in type H vessel formation, affecting the healing of bone defects. To test this hypothesis, we constructed a co‐culture system of HUVECs and osteoblasts. The effects of SIRT1 activation or inhibition on HUVEC angiogenic and osteogenic properties were investigated in the co‐culture system in vitro. Moreover, the effects of SIRT1 activity on type H vessels and osteogenesis in murine femur and mandible bone defects were also assessed. Collectively, our findings identify a novel and feasible target for enhancing bone repair via type H vessels.

## 2 METHODS

### 2.1 Cell culture

Dulbecco's modified Eagle medium (DMEM) supplemented with 10% foetal bovine serum (FBS; Gibco BRL, Grand Island, USA) was used to culture HUVECs in a 5% CO_2_ incubator at 37°C. Three‐day‐old C57BL/6 mice were used to isolate primary murine calvarial osteoblasts. In brief, the murine calvarial bone was cut into pieces, digested with trypsin‐ethylenediaminetetraacetic acid (EDTA) for 30 min, and incubated with 0.5% mg/mL collagenase I overnight at 37°C. The tissues were centrifuged at 1200 × *g* for 8 min, and the supernatant was discarded. The precipitate was re‐suspended and cultured in DMEM supplemented with 10% FBS.

After centrifugation, osteoblasts and HUVECs were digested and re‐suspended for the co‐culture system. With a total concentration of 3 × 10^5^ cells/well, the two cell types were mixed and seeded in 6‐well plates at HUVEC‐to‐osteoblast ratios of 5:1, 1:1, 1:5, and 1:10. By counting the number of reticular structures under the microscope after 3 or 7 days. The angiogenic potential of each co‐culture ratio was assessed, and the appropriate time and ratio were selected for subsequent angiogenesis experiments.

The co‐culture systems were routinely cultivated as previously reported for osteogenic differentiation.^23^ Osteogenic differentiation was induced using an induction media comprising complete DMEM, 50 g/mL ascorbic acid, 10 nM dexamethasone, and 10 mM beta‐glycerophosphate. The media was changed every 2 days. For alkaline phosphatase (ALP) staining (Alkaline Phosphatase Assay Kit, Beyotime, China), the cells were cultured in osteogenic media for 3 or 7 days; the appropriate time and proportion of the co‐culture systems were selected for subsequent osteogenesis experiments.

### 2.2 Cell proliferation and migration

HUVECs were seeded in 96‐well plates at 5 × 10^3^ cells/well for the Cell Counting Kit‐8 (CCK‐8) assay (APExBIO, Houston, Texas, USA). Gradient concentrations of SRT1720 (a specific SIRT1 agonist) or EX527 (a specific SIRT1 inhibitor)^24^ (SRT1720: 0, 0.5, 1, 2.5, 5, 7.5, and 10 μM; EX527: 0, 5, 10, 20, 30, 40, and 50 μM; APExBIO, Houston, Texas) were used. After 24 and 48 h, the cells were analysed using a CCK‐8 assay in accordance with the manufacturer's instructions.

To determine how SRT1720 or EX527 affect HUVEC migration, scratch wound experiments were carried out. In brief, 2 × 10^5^ cells/well were plated in 6‐well plates and incubated at 37°C until confluence was reached. To remove the detached cells, the monolayer was scraped and rinsed in phosphate‐buffered saline (PBS). The cells were then grown in serum‐free DMEM and supplemented with SRT1720 or EX527 at concentrations of 0, 0.5, 1, or 2.5 μM for SRT1720 and 0, 5, 10, or 20 μM for EX527. At 0 and 24 h, cell imaging was carried out by an inverted microscope (OLYMPUS, Japan).

Transwell assays were performed. Cells were seeded in the upper chamber at a density of 1 × 10^4^ per well. The lower chambers were filled with DMEM+10% FBS and upper chambers were filled with DMEM. After 24 h, the top chamber was gently removed and the cells were fixed with 4% paraformaldehyde and stained with 0.5% crystal violet dye.

### 2.3 Tube formation assay

In 48‐well plates, Matrigel (BD Biosciences, New Jersey, USA) was plated and allowed to gelate for 30 min at 37°C. HUVECs were added to polymerized Matrigel on plates treated with or without SRT1720 or EX527, at a density of 2 × 10^4^ cells/well. An inverted microscope (OLYMPUS, Japan) was used to monitor tube development after 6 h of incubation at 37°C. Using ImageJ software, the number of branches and total branch length were calculated.

### 2.4 RNA extraction, quantitative real‐time polymerase chain reaction analysis, and western blot analysis

TRIzol reagent (Invitrogen, USA) was used to extract total RNA from cells in accordance with the manufacturer's instructions. The PrimeScriptTM RT Reagent Kit with gDNA Eraser (Takara, Japan) was used to reverse‐transcribe RNA. SYBR‐Green 2X Master Mix (Bimake, China) was used to produce a quantitative real‐time polymerase chain reaction (qRT‐PCR). The primers used in this procedure are listed in Table S1.

The cells were lysed using RIPA lysis buffer (Solarbio, China) in preparation for western blotting. Proteins were isolated using polyacrylamide gel electrophoresis with sodium dodecyl sulphate before being transferred to polyvinylidene fluoride membranes. For immunoblotting, primary and secondary antibodies were utilized (Table S2). The manufacturer‐recommended dilution ratio was used for all antibodies. The cells were incubated with primary antibodies overnight at 4°C; secondary antibodies were then added for 1 h at room temperature and washed three times for 15–20 min each at the end of each incubation. Bands were visualized using an ECL kit (Abbkine, China).

### 2.5 HUVEC and osteoblast co‐culture

After the preliminary determination of appropriate cell ratios and exposure times, SRT1720 or EX527 was added to the co‐culture system at different proportions on Days 3 and 7. qRT‐PCR analysis of *Cd31*, *Emcn*, *Vegf*, and hypoxia‐inducible factor 1 subunit alpha (*Hif1a*) expression and western blot analysis of CD31, EMCN, VEGF, and HIF1A abundance were performed on Day 3 to determine the angiogenic capacity. ALP staining was performed on Day 7, and Alizarin red S (ARS; Sigma) staining was conducted on Day 14. qRT‐PCR analysis of *Alp*, *Runx2*, *Sp7*, and collagen type I alpha 1 chain (*Col1a1*) expression and western blot analysis of ALP, RUNX2, and SP7 abundance were performed to assess the osteogenic capacity of the co‐culture system on Day 7.

### 2.6 Immunofluorescence

The cells were fixed for 10 min in a 4% paraformaldehyde solution before immunofluorescence. They were then washed three times with PBS, treated with 0.5% Triton X‐100 for 15 min, and blocked with 1% bovine serum albumin (Solarbio, China) for 0.5 h. The cells were incubated with the CD31 primary antibody at 4°C overnight and subsequently incubated with the secondary antibody. The cell nuclei were stained with 4′,6‐diamidino‐2‐phenylindole.

### 2.7 Enzyme‐linked immunosorbent assay

The culture media from each group was collected and centrifuged at 3600 × *g* for 20 min to remove cell debris, and the supernatant was then transferred to fresh Eppendorf tubes for the enzyme‐linked immunosorbent assay (ELISA). The mouse Slit Guidance Ligand 3 (SLIT3) and human transforming growth factor beta (TGF‐β) ELISA kits (MEIMIAN, China) were used for the immunoassay. The absorbance value was measured for each well at 450 nm, and the concentration of SLIT3/TGF‐β in each group was determined using the standard curve.

### 2.8 Small interfering RNA transfection

A small interfering negative control and small interfering RNA (siRNA) for protein kinase B (AKT si‐AKT) were pre‐diluted in serum‐free Opti‐MEM with 5% EndoFectin (Gene Pharma, China). Table S3 provides a list of the siRNA sequences. Cells were transfected with the siRNAs for 24 h at 37°C. Subsequently, the cells were washed and the media was replaced. After 3 days, CD31 immunofluorescence was carried out, and ALP staining was performed on Day 7 to assess osteogenic differentiation. Western blot and qRT‐PCR studies were carried out, as previously described.

### 2.9 Mice bone defect models

Total of 60 male 8‐week‐old C57BL/6 mice were used and divided into three groups (Con, SRT1720 and EX527) A dental engine (Wallingford, USA) was used to create a monolayer cortical bone defect in the femur or mandible after anaesthesia (1 mm in diameter). Following surgery, intraperitoneal injections of SRT1720 and EX527 (20 mg/kg/day for SRT1720 and 2 mg/kg/day for EX527) were administered; the control group was treated with the same amount of PBS. One or 2 weeks following treatment, each group was euthanized by cervical dislocation. The bone tissue was isolated via treatment with 4% paraformaldehyde solution for 6 h, followed by treatment with 70% ethanol until the microcomputed tomography (micro‐CT) scanning was performed.

### 2.10 Micro‐CT scanning and histology staining

Micro‐CT (u‐CT80, SCANCO, Zurich Switzerland) was used to scan and evaluate femur samples from post‐treatment mice. The samples were scanned using micro‐CT with an integration time of 300 ms and operating voltage and current of 70 kV and 200 A, respectively. The area of concern was a 1‐mm thick femur bone defect protruding from the bone surface. The bone volume‐to‐total volume ratio (BV/TV), trabecular separation (Tb.Sp), trabecular number (Tb.N), and trabecular thickness (Tb.Th) were among the metrics evaluated.

As previously reported, following micro‐CT analysis, samples from the femur and mandible were histologically stained.^23^ Decalcification was achieved by incubating the samples in 15% EDTA at 4°C for 2–3 weeks, with the EDTA changed every 2 days. Bone tissue sections were subjected to immunofluorescence staining to visualize CD31, EMCN, ALP, RUNX2, and HIF1A expression. Tissues were also stained with Masson's trichrome to visualize connective tissues, and haematoxylin and eosin (HE) to visualize cellular and tissue structure details. Additionally, immunohistochemical analysis was performed for p‐AKT. The staining results were measured using ImageJ.

### 2.11 Statistical analysis

The quantitative data are presented as mean and standard deviation (SD). A one‐way analysis of variance (ANOVA) was used to compare the groups. The experimental findings were analysed using IBM SPSS Statistics software (version 20.0; IBM Corp., Armonk, NY, USA). The threshold for significance was set at *p* < 0.05.

## 3 RESULTS

### 3.1 SIRT1 activation enhances the proliferation, migration, and angiogenesis of HUVECs

**FIGURE 1:**
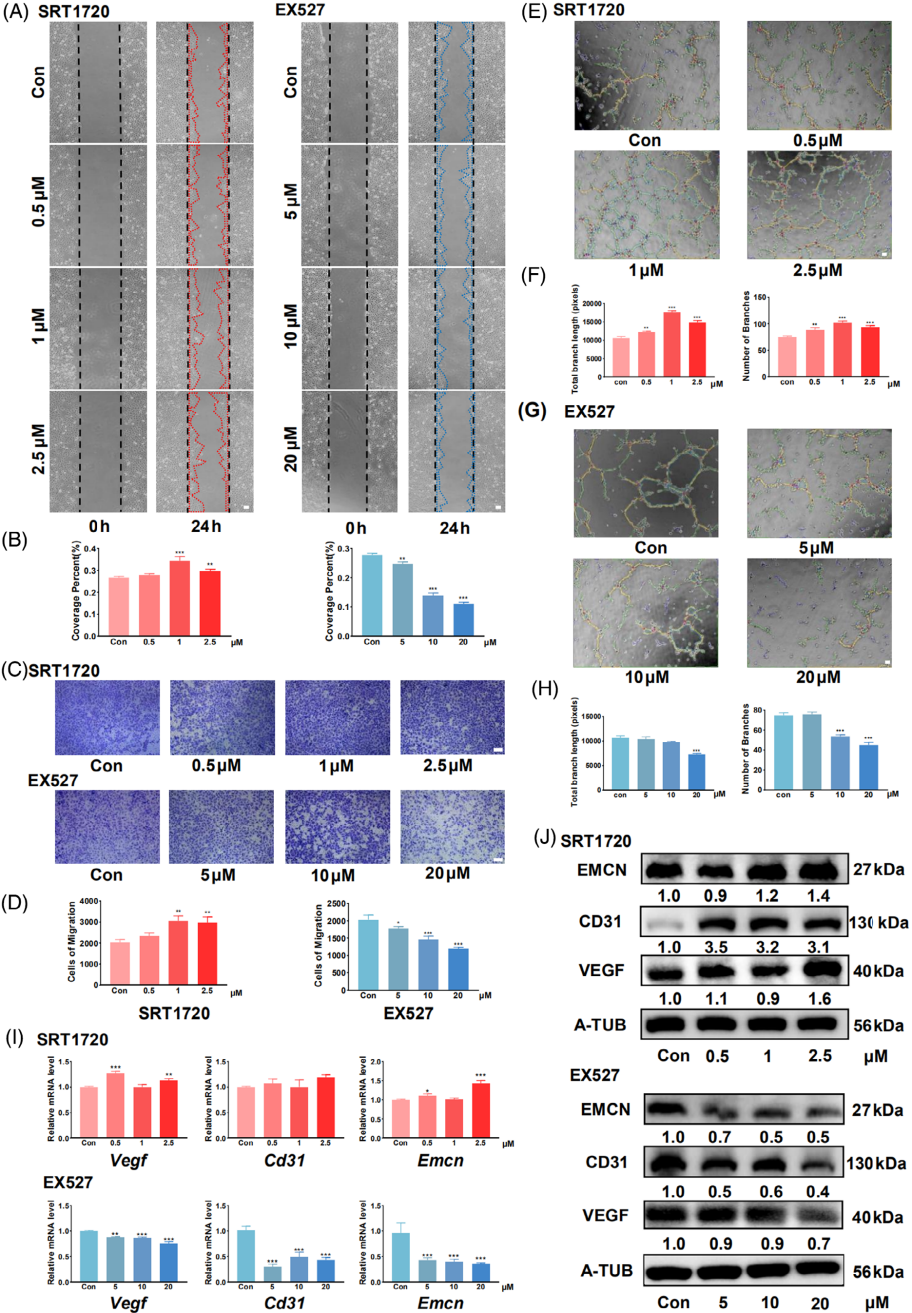
SIRT1 activation promotes the angiogenic capacity of HUVECs. (A) Treatment with different concentrations of SRT1720 promotes scratch healing in HUVECs, and treatment with EX527 causes the opposite effect. (B) Quantitative analysis of scratch healing assay in the SRT1720 and EX527 treatment groups. (C) SRT1720 treatment promotes migration and EX527 treatment inhibits migration, as demonstrated in the Transwell assay. (D) Quantitative analysis of the different treatment groups in the Transwell assay. (E) Results of the tube formation assay following SRT1720 treatment. (F) Quantitative analysis of total branch length and number of branches in the SRT1720‐treated group. (G) Results of the tube formation assay following treatment with EX527. (H) Quantitative analysis of total branch length and number of branches in the EX527‐treated groups. (I) qRT‐PCR results of factors involved in the angiogenic capacity of HUVECs after treatment with SRT1720 or EX527. (J) Western blot results of HUVECs after treatment with SRT1720 or EX527. Scale bar = 500 μm. All data are presented as the mean ± SD, *n* = 3. *p < 0.05 **p < 0.01 ***p < 0.001 relative to the control group. Differences were analysed using one‐way ANOVA. ANOVA, analysis of variance; HUVEC, human umbilical vein endothelial cells; qRT‐PCR, quantitative real‐time polymerase chain reaction; SD, standard deviation; SIRT1, sirtuin 1.

The CCK8 assay revealed that treatment with 0.5, 1, or 2.5 μM SRT1720 promoted HUVEC proliferation, whereas treatment with 5, 10, or 20 μM EX527 had the opposite effect (Figure S1A). The findings of the scratch wound‐healing experiment showed that SIRT1 activation increased HUVEC migration, whereas SIRT1 inhibition had the opposite effect (Figure 1A). Quantitative analysis results indicated a significant increase in cell migration with 1 or 2.5 μM SRT1720 and decrease following treatment with 5, 10, or 20 μM EX527 (Figure 1B). The Transwell migration assay yielded similar results (Figure 1C,D). Furthermore, HUVECs with activated SIRT1 formed more tubes and branches on Matrigel, whereas inhibition of SIRT1 weakened this capacity (Figure 1E–H). The expression levels of CD31, EMCN, and VEGF were also positively correlated with SIRT1 activation (Figures 1I,J and S4). Additionally, a favourable correlation was observed between the levels of SIRT1 activity and CD31, EMCN, and VEGF expression.

### 3.2 SIRT1 activation promotes the angiogenic and osteogenic abilities of the HUVEC/osteoblast co‐culture system in vitro

**FIGURE 2:**
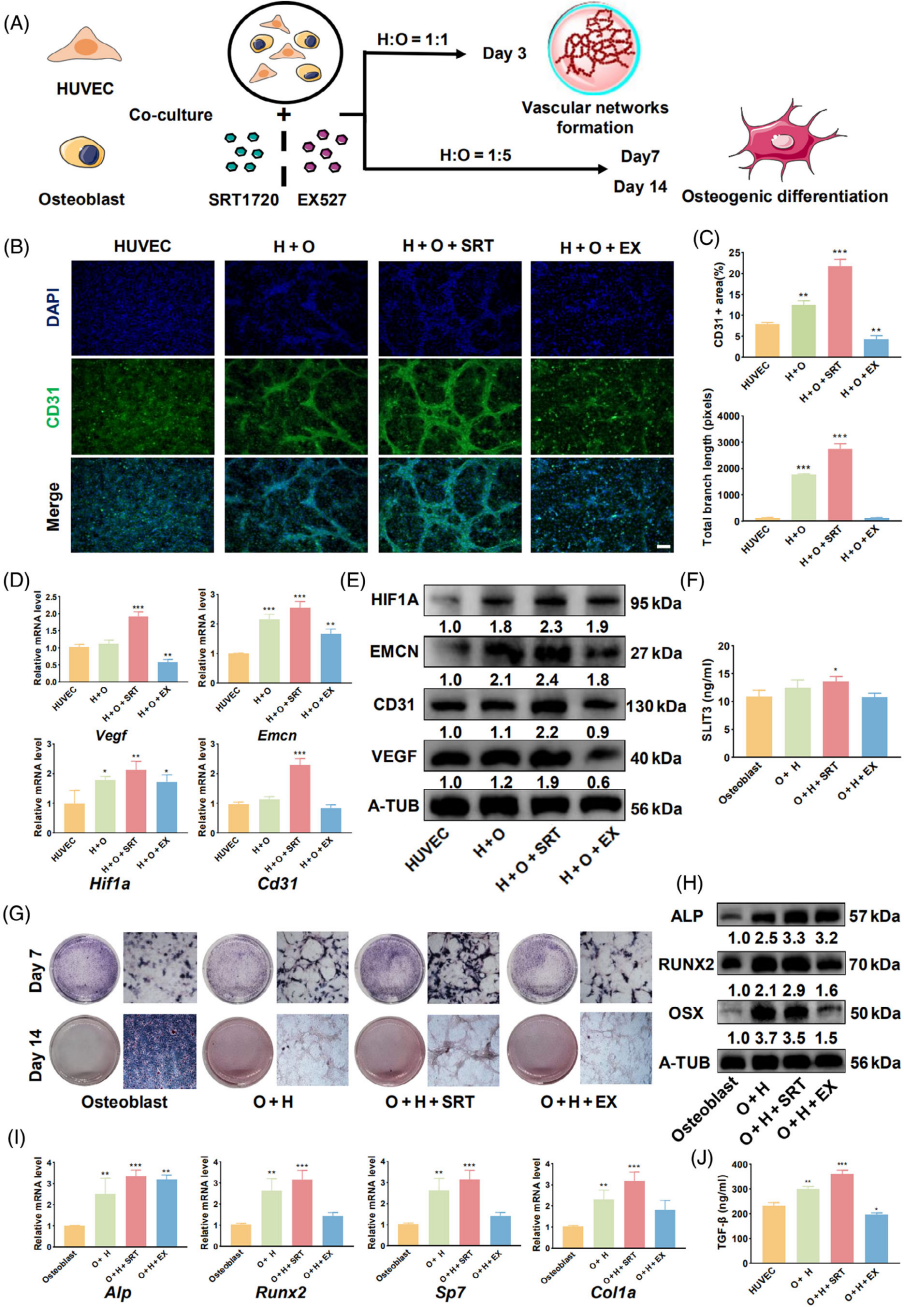
SIRT1 activation enhances the angiogenic and osteogenic capacities of the HUVEC/osteoblast co‐culture system. (A) The co‐culture system was constructed using different cell ratios and incubation times to investigate the effects of SIRT1 activation or inhibition on the osteogenic and angiogenic abilities of the system. (B) Immunofluorescence staining demonstrating that activating SIRT1 in the co‐culture system enhances vascular formation in HUVECs, whereas inhibiting SIRT1 reduces it. (C) Quantitative analysis of CD31‐positive cell area and total branch length. (D) qRT‐PCR analysis of angiogenic genes in the co‐culture system. (E) Western blot analysis of angiogenic proteins in the co‐culture system. (F) ELISA demonstrating SLIT3 secretion from osteoblasts in the co‐culture system. (G) ALP and ARS staining of the co‐culture system at different time points. (H) qRT‐PCR analysis of osteogenic genes in the co‐culture system at 7 days. (I) Western blot analysis of osteogenic proteins in the co‐culture system at 7 days. (J) ELISA demonstrating TGF‐β secretion from HUVECs in the co‐culture system. Scale bar = 500 μm. All data are presented as the mean ± SD, *n* = 3. *p < 0.05, **p < 0.01, ***p < 0.001 relative to the control group. Differences were analysed using one‐way ANOVA. ALP, alkaline phosphatase; ANOVA, analysis of variance; ARS, Alizarin red S; CD31, cluster of differentiation 31; ELISA, enzyme‐linked immunoassay; H or HUVEC, human umbilical vein endothelial cells; O, osteoblast; qRT‐PCR, quantitative real‐time polymerase chain reaction; SD, standard deviation; SIRT1, sirtuin 1; SLIT3, slit guidance ligand 3; TGF‐β, transforming growth factor beta.

Based on our preliminary experiments, appropriate cell ratios and exposure times were selected for further experiments (Figure S2A,B). Subsequently, SRT1720 and EX527 treatments were used to examine the effects of SIRT1 activation or inhibition on the angiogenic and osteogenic abilities of the cells in our co‐culture system (Figure 2A).

Regarding angiogenesis, we observed that direct co‐culture led to the spontaneous formation of a vascular network, even in the absence of Matrigel, which has been previously reported in the co‐cultures of HUVECs and human bone marrow‐derived MSCs^25^ (Figure S2A). Immunofluorescence staining of the network revealed high expression of CD31, indicating that the network was primarily formed by HUVECs. SIRT1 activation significantly enhanced the formation of vascular networks in the co‐culture system, whereas SIRT1 inhibition reduced angiogenesis (Figure 2B,C). The expression of angiogenic genes and proteins (VEGF, CD31, EMCN, and HIF1A,) also exhibited a significant increase upon SIRT1 activation (Figures 2D,E and S5). Furthermore, secretion of the angiogenic factor SLIT3 into the media of the co‐culture system was significantly increased (Figure 2F).

The results of the ALP and ARS staining experiments indicated that SIRT1 activation significantly enhanced the osteogenic ability of the co‐culture system (Figure 2G). Additionally, the expression levels of osteogenic factors (ALP, RUNX2, OSX, and COL1A) increased, whereas SIRT1 inhibition suppressed the osteogenic capacity of the system (Figures 2H,I and S5). In addition, the concentration of TGF‐β derived from HUVECs in the medium was significantly increased, and SIRT1 activation further amplified this effect (Figure 2J). However, no notable enhancement in the osteogenic ability of osteoblasts was observed by either SIRT1 activation or inhibition (Figure S3A–C).

### 3.3 Phosphatidylinositol 3‐kinase/AKT/forkhead box O1 pathway mediates the effects of SIRT1 in the HUVEC/osteoblast co‐culture system

**FIGURE 3:**
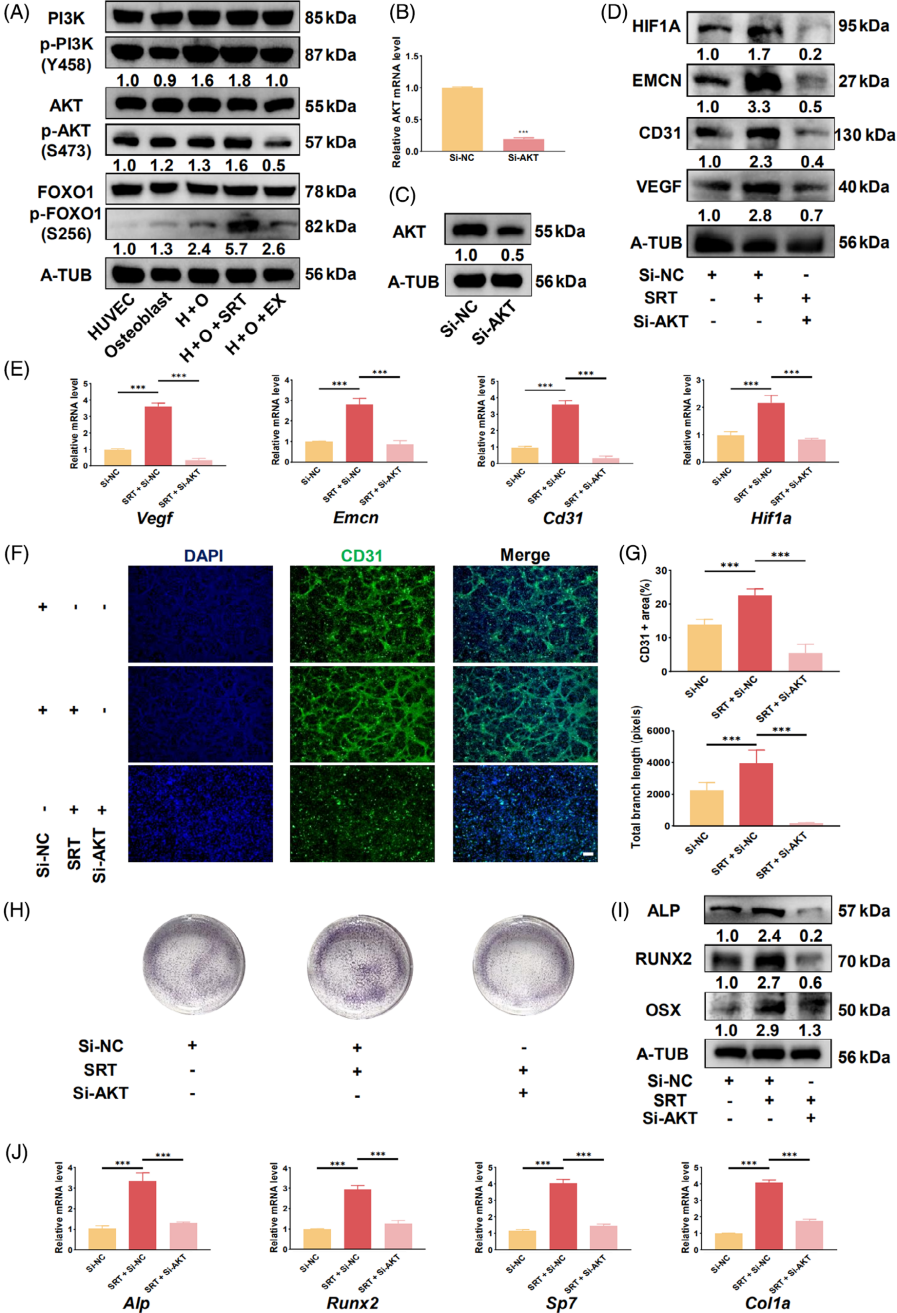
The PI3K/AKT/FOXO1 signalling pathway in HUVECs mediates SIRT1 angiogenic and osteogenic effects in co‐culture. (A) SIRT1 activation promotes the phosphorylation of the PI3K/AKT/FOXO1 signalling pathway in the co‐culture system. (B) qRT‐PCR analysis showing that si‐AKT transfection significantly reduces *Akt* expression in HUVECs within the co‐culture system. (C) Western blot analysis showing a reduction of AKT expression in the co‐culture system following si‐AKT transfection. (D) si‐AKT transfection abolishes the enhancing effect of SIRT1 activation on the expression of angiogenesis‐related proteins. (E) qRT‐PCR results illustrating the expression of type H vessel‐related genes in the co‐culture system after si‐AKT transfection. (F) Immunofluorescence staining demonstrating that si‐AKT transfection inhibits the vascular formation induced by SIRT1 activation in the co‐culture system. (G) Quantitative analysis of the CD31‐positive area and total branch length after si‐AKT transfection. (H) ALP staining revealing that si‐AKT transfection attenuates the promoting effect of SIRT1 on the osteogenic capacity of the co‐culture system. (I) Expression of osteogenesis‐related proteins in the co‐culture system after si‐AKT transfection. (J) Expression of osteogenesis‐related genes in the co‐culture system after si‐AKT transfection. Scale bar = 500 μm. All data are presented as the mean ± SD, *n* = 3. *p < 0.05, **p < 0.01, ***p < 0.001 relative to different groups. Differences were analysed using one‐way ANOVA. AKT, protein kinase B; ALP, alkaline phosphatase; ANOVA, analysis of variance; CD31, cluster of differentiation 31; FOXO1, forkhead box O1; HUVEC, human umbilical vein endothelial cells; PI3K, phosphatidylinositol 3‐kinase; qRT‐PCR, quantitative real‐time polymerase chain reaction; SD, standard deviation; siRNA, short interfering RNA; SIRT1, sirtuin 1.

Western blotting results showed that the co‐culture increased phosphorylation levels of proteins associated with the phosphatidylinositol 3‐kinase (PI3K)/AKT/forkhead box O1 (FOXO1) pathway, which was further enhanced or weakened upon SIRT1 activation or inhibition, respectively (Figures 3A and S6). After siRNA transfection, AKT expression significantly decreased in the co‐culture system (Figures 3B,C and S6). Moreover, angiogenesis‐related genes and proteins also showed a noticeable reduction in expression (Figures 3D,E and S6). The number of vascular networks in the co‐culture system also decreased (Figure 3F,G). Additionally, ALP staining showed that the osteogenic capacity of the co‐culture system decreased following 7 days of si‐AKT transfection (Figure 3H); the expression of genes and proteins related to osteogenesis significantly decreased (Figure 3I,J and S6).

### 3.4 SIRT1 activation promotes bone healing in femur and mandible bone defects

**FIGURE 4:**
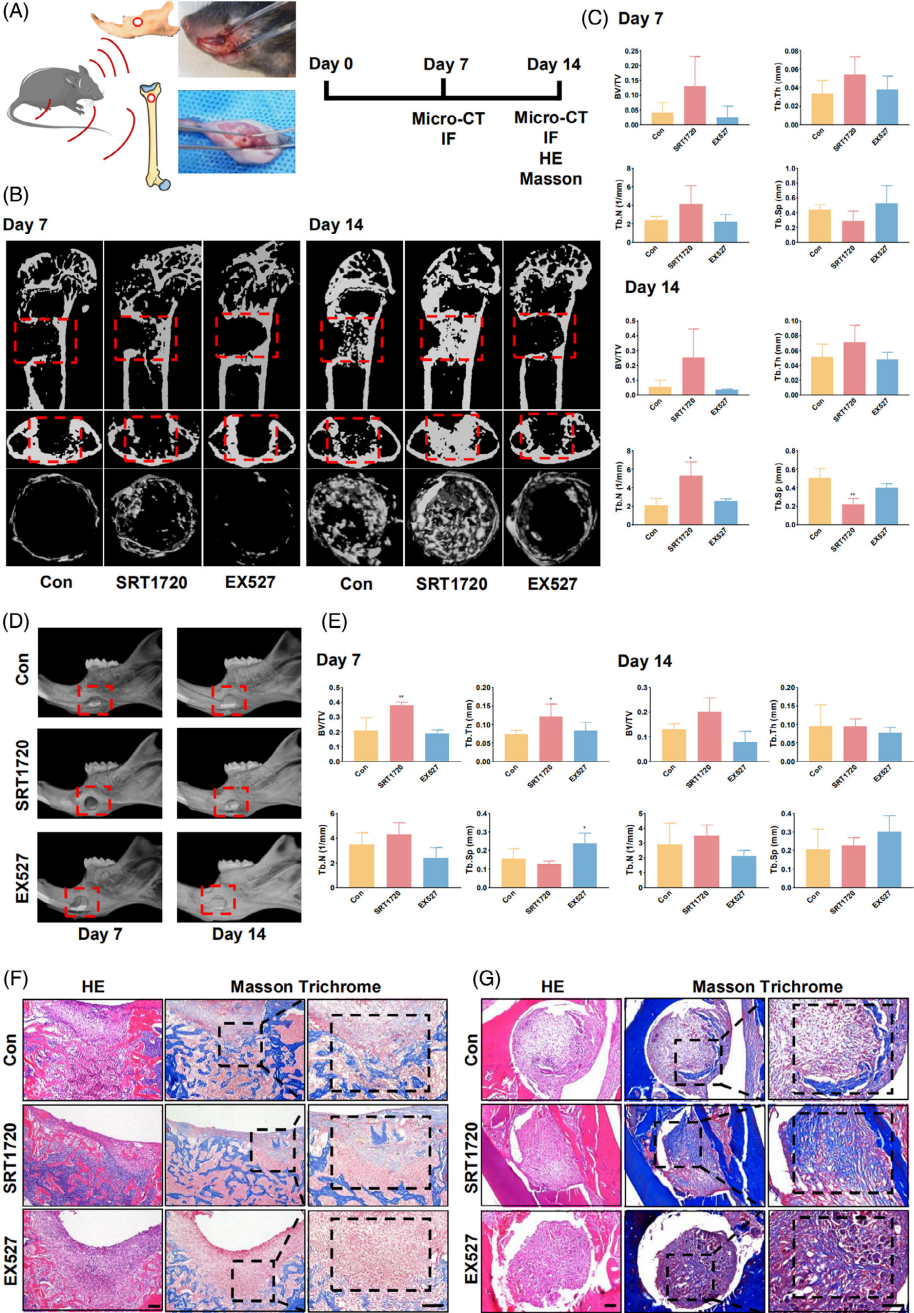
SIRT1 activation promotes bone healing in femur and mandible bone defects. (A) A schematic diagram illustrates the construction of mouse femoral and mandibular bone defect models. (B) Micro‐CT images depicting the progression of femoral bone defects at 7 and 14 days in 8‐week‐old male mice (The red box indicates the healing of the bone defects). (C) Quantitative analysis of femoral bone defect healing process using BV/TV, Tb.N, Tb.Sp, and Tb.Th. (D) Micro‐CT reconstructed images depicting the progression of mandibular bone defects at 7 and 14 days in 8‐week‐old male mice (The red box indicates the healing of the bone defects). (E) Quantitative analysis of mandibular bone defect healing process. (F) The results of HE and Masson's trichrome staining of femoral bone defects (The black box indicates new bone formation in the bone defects). (G) The results of HE and Masson's trichrome staining of mandibular bone defects (The black box indicates new bone formation in the bone defects). Scale bar = 100 μm. All data are presented as the mean ± SD, *n* = 5. *p < 0.05, **p < 0.01, ***p < 0.001 relative to the control group. Differences were analysed using one‐way ANOVA. ANOVA, analysis of variance; BV/TV, bone volume to total volume; HE, haematoxylin and eosin; micro‐CT, microcomputed tomography; SD, standard deviation; SIRT1, sirtuin 1; Tb.N, trabecular number; Tb.Sp, trabecular separation; Tb.Th, trabecular thickness.

To provide further evidence supporting the role of SIRT1 in bone healing, bone defects in the femur or mandible of mice were induced, followed by treatment with SRT1720 or EX527 (Figure 4A). Notably, micro‐CT results demonstrated that SIRT1 activation significantly promoted bone defect healing at various time points, whereas the inhibition of SIRT1 slowed this process in the femur and mandible (Figure 4B–E). For femurs, quantitative analysis revealed a significant difference only in the Tb.N and Tb.Sp bone healing measurements in the SRT1720 treatment group at 14 days, with no significant differences among the other measurements (Figure 4C). BV/TV, Tb.Sp, and Tb.Th in the SRT1720 treatment group at 14 days also showed significant differences in the mandible, whereas differences in the other indicators were not observed (Figure 4E). Consistent with these findings, HE and Masson's trichrome staining of bone defects indicated that SRT1720 treatment facilitated new bone formation and maturation (Figure 4F,G).

### 3.5 SIRT1 activation enhances the coupling of type H vessels and osteogenic/angiogenic factors in bone defects through the PI3K/AKT/FOXO1 pathway

**FIGURE 5:**
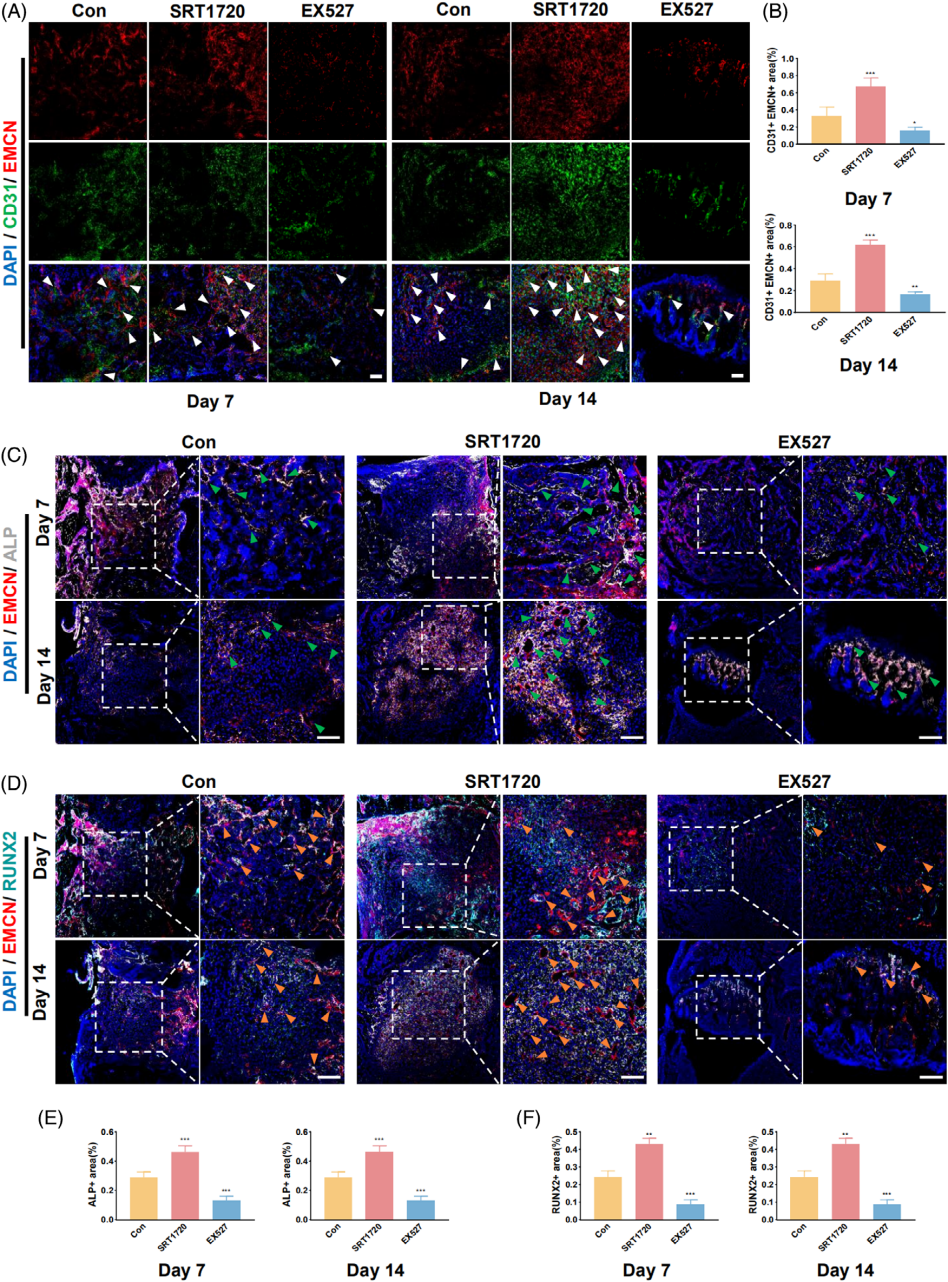
SIRT1 activation promotes type H vessel formation and osteogenic factor expression in femoral bone defects. (A) Immunofluorescence staining revealing the formation of type H vessels at 7 and 14 days in femoral bone defects in 8‐week‐old mice (The white arrow indicates both CD31‐ and EMCN‐positive cells). (B) Quantitative analysis of CD31‐ and EMCN‐positive areas at 7 and 14 days. (C) Expression of ALP and EMCN at 7 and 14 days in femoral bone defects (The green arrow indicates both ALP‐ and EMCN‐positive cells). (D) Expression of RUNX2 and EMCN at 7 and 14 days in femoral bone defects (The orange arrow indicates both RUNX2‐ and EMCN‐positive cells). (E) Quantitative analysis of ALP‐positive areas at 7 and 14 days. (F) Quantitative analysis of RUNX2 positive areas at 7 and 14 days. Scale bar = 100 μm. All data are presented as the mean ± SD, *n* = 5. *p < 0.05, **p < 0.01, ***p < 0.001 relative to the control group. Differences were analysed using one‐way ANOVA. ALP, alkaline phosphatase; ANOVA, analysis of variance; CD31, cluster of differentiation 31; EMCN, endomucin; micro‐CT, microcomputed tomography; RUNX2, runt‐related transcription factor 2; SD, standard deviation; SIRT1, sirtuin 1.

Immunofluorescence staining revealed that SRT1720 therapy considerably promoted the creation of type H vasculature in femur bone defects, whereas EX527 treatment impeded this process (Figure 5A). Between Days 7 and 14, quantitative analysis revealed a substantial variation in the expression of type H vascular markers (Figure 5B). Regarding osteogenic factors co‐expressed with type H vessels in the vicinity of a bone defect, such as ALP and RUNX2, SIRT1 activation significantly increased their expression, whereas SIRT1 inhibition had the opposite effect (Figure 5C–F).

**FIGURE 6:**
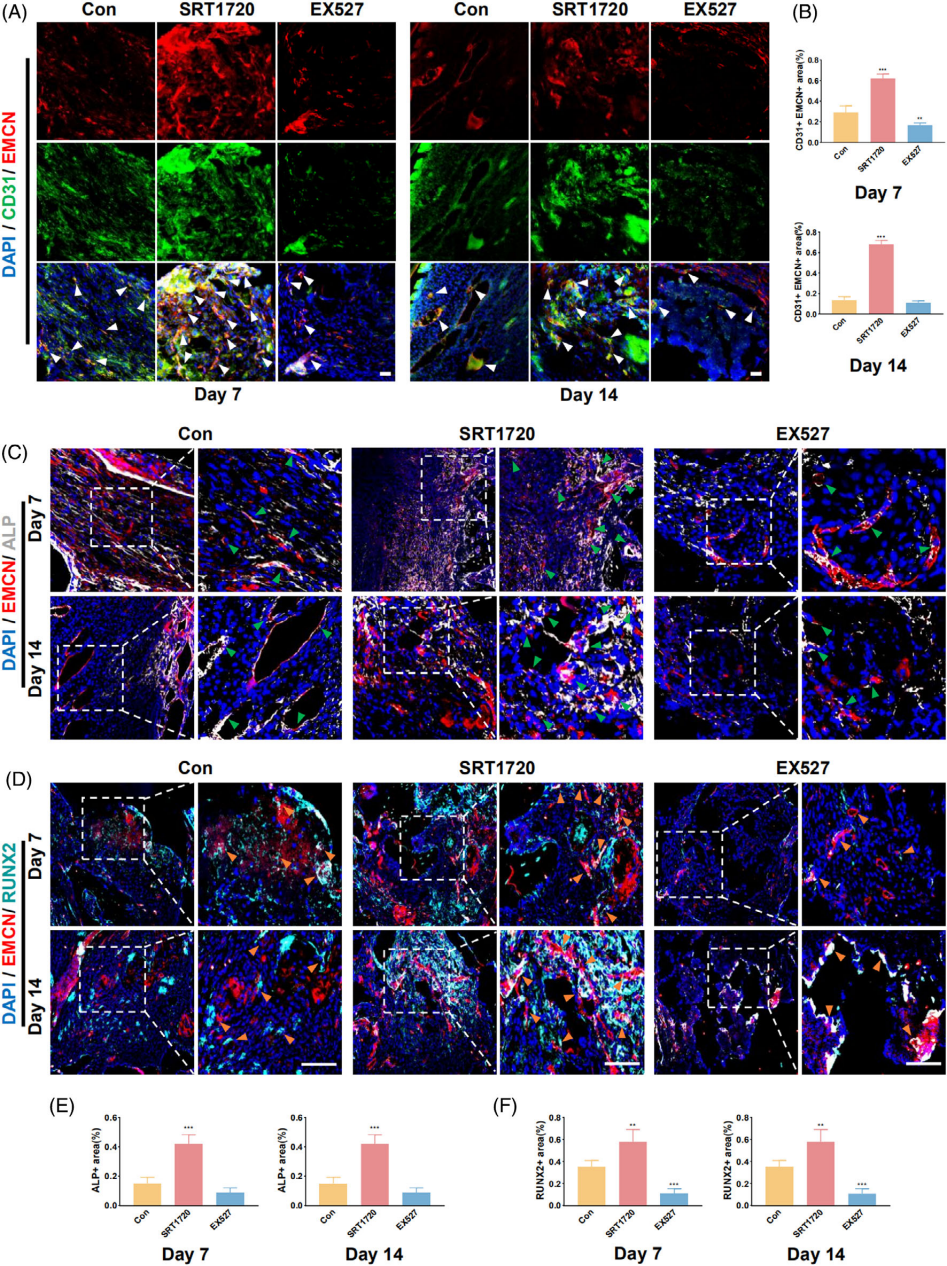
SIRT1 activation promotes type H vessel formation and osteogenic factor expression in mandibular bone defects. (A) Immunofluorescence staining revealing the formation of type H vessels at 7 and 14 days in mandibular bone defects in 8‐week‐old mice (The white arrow indicates both CD31‐ and EMCN‐positive cells). (B) Quantitative analysis of CD31‐ and EMCN‐positive areas at 7 and 14 days. (C) Expression of ALP and EMCN at 7 and 14 days in mandibular bone defects (the green arrow indicates both ALP‐ and EMCN‐positive cells). (D) Expression of RUNX2 and EMCN at 7 and 14 days in mandibular bone defects (The orange arrow indicates both RUNX2‐ and EMCN‐positive cells). (E) Quantitative analysis of ALP‐positive areas at 7 and 14 days. (F) Quantitative analysis of RUNX2 positive areas at 7 and 14 days. Scale bar = 100 μm. All data are presented as the mean ± SD, *n* = 5. *p < 0.05, **p < 0.01, ***p < 0.001 relative to the control group. Differences were analysed using one‐way ANOVA. ALP, alkaline phosphatase; ANOVA, analysis of variance; CD31, cluster of differentiation 31; EMCN, endomucin; RUNX2, runt‐related transcription factor 2; SD, standard deviation; SIRT1, sirtuin 1.

For the mandibular defects, SIRT1 activation also exhibited a positive correlation with type H vessels (Figure 6A,B). Additionally, the expression of ALP and RUNX2 was significantly increased in the SRT1720 treatment group in mandibular defects. However, in the EX527 treatment group, significant differences were observed in RUNX2 expression, but not in that of ALP (Figure 6C–F).

**FIGURE 7:**
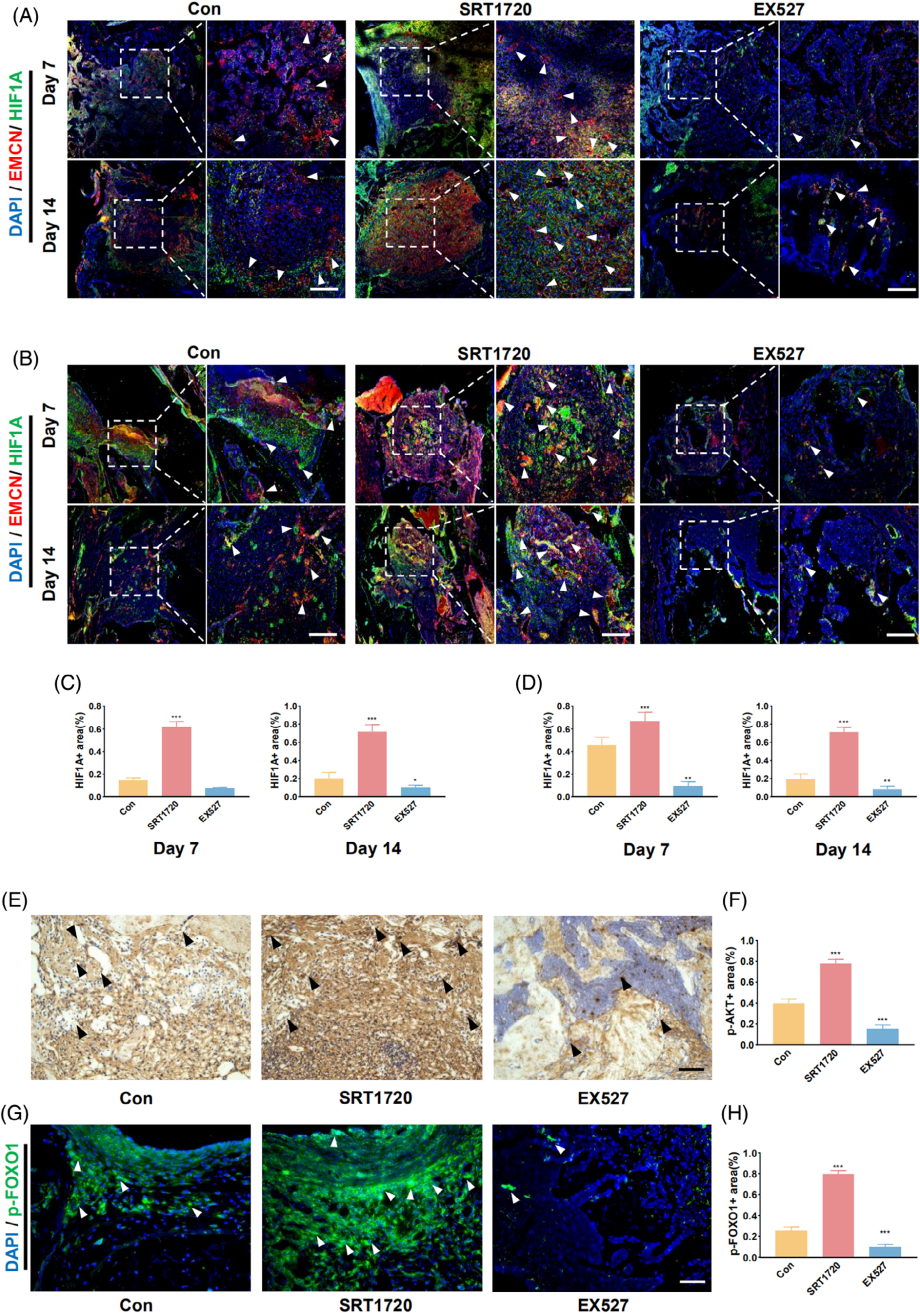
SIRT1 activation promotes HIF1A expression in femur and mandible bone defects through the AKT‐related pathway. (A) Immunofluorescence staining revealing the expression of EMCN and HIF1A at 7 and 14 days in mouse femoral bone defects (The white arrow indicates the both HIF1A‐ and EMCN‐positive cells). (B) Expression of EMCN and HIF1A at 7 and 14 days in mouse mandibular bone defects (The white arrow indicates both HIF1A‐ and EMCN‐positive cells). (C) Quantitative analysis of HIF1A expression at 7 and 14 days in the femoral bone defects. (D) Quantitative analysis of HIF1A expression at 7 and 14 days in mandibular bone defects. (E) Immunohistochemical staining of p‐AKT in the bone defective area at 7 days (The black arrow indicates p‐AKT positive cells). (F) Quantitative analysis of p‐AKT immunohistochemical staining in bone defects. (G) Immunofluorescence staining of p‐FOXO1 in the bone defective area at 7 days (The white arrow indicates p‐FOXO1 positive cells). (H) Quantitative analysis of p‐FOXO1 immunofluorescence staining in bone defects. Scale bar = 100 μm. All data are presented as the mean ± SD, *n* = 5. *p < 0.05 **p < 0.01 ***p < 0.001 relative to the control group. Differences were analysed using one‐way ANOVA. AKT, protein kinase B; ANOVA, analysis of variance; EMCN, endomucin; HIF1A, hypoxia inducible factor 1 subunit alpha; SD, standard deviation; SIRT1, sirtuin 1.

HIF1A expression is closely associated with the formation of type H vessels. The activation of SIRT1 upregulated HIF1A expression at the site of the bone defects (Figure 7A,B). In the femur defects, quantitative analysis showed a favourable connection between HIF1A expression and SIRT1 activity (Figure 7C). In mandibular defects, HIF1A expression was higher during the early stage and decreased in the later stage, consistent with the changes observed in type H vessel formation (Figure 7D). Additionally, immunohistochemistry and immunofluorescent staining showed that SIRT1 activation significantly increased the expression of p‐AKT and p‐FOXO1 in bone defects (Figure 7E–H).

## 4 DISCUSSION

The function of type H vasculature, distinguished by their high expression of CD31 and EMCN, in bone modelling and re‐modelling has attracted increasing attention.^7^ Emerging research has shown that type H vessels generated during bone regeneration exhibit a significantly different morphology than in structured metaphyseal regions, displaying a more chaotic and disorganized pattern.^26^, ^27^ This finding raises the possibility that type H arteries aid in bone repair.^28^ Type H vessels are regulated by a number of bodily substances, including SLIT3 and platelet‐derived growth factor BB (PDGF‐BB).^9^, ^29^ Although the involvement of SIRT1 in regulating vascular generation has been confirmed, its precise role in angiogenesis‐osteogenesis coupling remains elusive.^30^, ^31^ To further evaluate SIRT1's capacity to control bone regeneration, this work investigated the regulatory function of SIRT1 in the development of type H vessels.

In this study, SIRT1 activation was conducive to the ability of HUVECs to generate vasculature, which is consistent with most previous findings.^32^, ^33^ By altering the expression of numerous angiogenesis‐promoting proteins, including VEGF and HIF1A, SIRT1 activation improves the capacity of HUVECs to produce blood vessels.^34^ Additionally, SIRT1 activation enhances CD31 and EMCN expression at the gene and protein levels, two components specifically linked to type H vessels. Remarkably, we observed a disparity between the mRNA and protein levels regarding the increase of CD31. While the mRNA level showed a less pronounced change, the protein level exhibited a more substantial increase. This phenomenon suggests the involvement of post‐transcriptional modifications and translational efficiency as contributing factors. Moreover, it is plausible that SIRT1 may have a relevant impact on protein stability, further influencing the observed results. Additionally, it is worth noting that SRT1720, as a specific activator of the SIRT1 pathway, might have a more pronounced effect on protein expression compared to mRNA levels. This could be attributed to the involvement of additional regulatory mechanisms, such as protein stability or post‐translational modifications, which can influence protein abundance independently of mRNA expression.

These findings suggest that SIRT1 likely plays a regulatory role in type H vessel formation; however, further confirmation via bone microenvironment stimulation is warranted.

Although osteoblasts have been predominantly studied for their role in promoting bone tissue mineralization during development,^35^, ^36^, ^37^ their specific contributions at the site of bone defects during the healing process have not been extensively reported. In this study, we developed a co‐culture approach involving osteoblasts and HUVECs in varying proportions to investigate their intricate interactions. We conducted experiments using a direct co‐culture method, which enables direct contact between different cells and facilitates a more effective exploration of cell communication, including cell junctions that cannot be achieved with indirect co‐culture methods like conditioned media and Transwell. However, as observed in our experiments, the direct co‐culture is significantly influenced by the ratio of different cells, making the selection of an appropriate cell ratio crucial. Therefore, indirect co‐culture methods may offer higher stability and greater significance in studying the effects of small molecular substances secreted by cells in further studies. As expected, osteoblasts induced the spontaneous formation of a vascular network structure by HUVECs in the co‐culture system. Furthermore, SIRT1 activation further enhanced vascular formation and osteogenic processes within the co‐culture system, whereas SIRT1 inhibition significantly weakened these processes. Interestingly, SIRT1 activation did not affect the osteogenic ability of osteoblasts. This contradicts earlier research results that suggested a link between SIRT1 activity and the osteogenic activity of BMSCs.^38^, ^39^ For instance, the administration of resveratrol to anorexic mice improves bone formation in the tibia and reduces adipose tissue production in the bone marrow.^40^ Mice with an MSC‐specific SIRT1 knockout exhibit a significant reduction in cortical bone and trabecular bone volume.^41^ Hence, the regulatory role of SIRT1 in BMSCs differs from that in osteoblasts. In particular, the regulatory effect of SIRT1 on osteoblasts appears to be more dependent on the presence of HUVECs within the co‐culture system, highlighting a critical distinction between the involvement of osteoblasts and BMSCs in the bone regeneration process.

Our results also indicate the involvement of the PI3K/AKT/FOXO1 pathway in the coupling of vascular formation and osteogenesis within the co‐culture system. The activation or inhibition of SIRT1 can enhance or weaken the phosphorylation of this pathway, respectively, suggesting that SIRT1 may manipulate this pathway to promote bone healing. Indeed, the PI3K/AKT signalling pathway is crucial for vascular growth and tumour angiogenesis.^42^, ^43^ Activated AKT stimulates nitric oxide synthase, which participates in vascular function, dilation, remodelling, and neovascularization.^44^ It also leads to increased expression of HIF1A, thus, regulating the expression of VEGF and other angiogenic factors to promote vascular formation.^45^, ^46^ Additionally, our findings agree with those indicating that the transcription factor FOXO1—a downstream regulator of the PI3K/AKT signalling pathway—participates in vascular formation.^47^ In our co‐culture system, AKT knockdown in HUVECs reduced the promotion of vascular formation and osteogenesis mediated by SIRT1 activation. This finding further supports the notion that SIRT1 regulates the osteogenic process of osteoblasts by mediating the PI3K/AKT/FOXO1 signalling pathway in HUVECs. However, others have shown that SIRT1 participates directly in the control of FOXO1^48^, ^49^; therefore, further research is required to determine whether this process also occurs during bone healing.

We created mice models of femur and mandible bone defects to further explore the possible function of SIRT1 in bone defect repair. Through micro‐CT scanning and histomorphological analysis of bone defects at different locations, we observed similar outcomes. That is, activation of SIRT1 increased the generation of type H vessels within bone defects and significantly promoted the process of bone regeneration, whereas inhibition of SIRT1 resulted in the opposite effect. Immunofluorescence staining revealed a significant positive correlation between an increase in ALP‐ and RUNX2‐positive cells and the quantity of type H vasculature. Additionally, HIF1A is closely correlated with the abundance of type H vessels during bone healing, and is highly sensitive to hypoxic microenvironments, enhancing vascular generation and improving local hypoxic conditions.^50^ Given that bone defects are commonly present in hypoxic environments, the process of vascular generation becomes crucial for bone healing, surpassing physiological bone metabolism.^51^, ^52^, ^53^ In addition, our results showed that mandibular defects expressed higher levels of HIF1A than femoral defects; this phenomenon was more significant at Day 7. This may indicate that the early hypoxic microenvironment is more evident in mandibular defects, which can be significantly improved by HIF1A, representing a potential difference in the healing processes of femur and mandible bone defects. Furthermore, immunohistochemistry analysis indicated the involvement of p‐AKT in the regulation of bone defect healing by SIRT1. This further demonstrates the importance of the SIRT1‐regulated PI3K/AKT/FOXO1 pathway in bone repair.

Although we investigated the effects of SIRT1 activation on type H vessels using an in vitro co‐culture system and bone defect models, certain unresolved issues remain. For instance, it remains unclear whether SIRT1 activation has consistent effects on osteoblasts in femur and mandible bone defects and whether the regulatory role of SIRT1 exists in different disease states, such as osteoporosis and inflammation. In addition, further investigations are warranted to determine whether the reported effects of SIRT1 on osteogenesis are achieved through indirect regulation by ECs.

## 5 CONCLUSIONS

This research offers important insights into the function of SIRT1 in the development of type H vessels and bone repair. It also provides more experimental evidence for the clinical application of SIRT1 activator in promoting bone healing. However, to fully elucidate the underlying mechanisms of these processes and investigate the therapeutic potential of SIRT1 in clinical settings, further studies are necessary.

## AUTHOR CONTRIBUTIONS

ZL, SL, and EL conceived the study. ZL, SL, HL, BL, and YL performed most of the experiments. ZL, SL, HL, and BL contributed to the analysis of the data. ZL, SL, HL, BL, YL, and EL discussed the results and wrote the article. All authors read and approved the final article.

## FUNDING INFORMATION

This study was supported by the National Natural Science Foundation of China (82370932 and 81970917); the Program of Science and Technology Department of Sichuan Province (23ZYZYTS0103); Research and Develop Program, West China Hospital of Stomatology Sichuan University (RD‐03‐202102); and Natural Science Foundation of Sichuan Province (2023NSFSC1512).

## CONFLICT OF INTEREST STATEMENT

The authors declare that they have no conflicts of interest.

## Supporting information


**FIGURE S1.** SIRT1 activation promotes the proliferation of HUVECs. (A) Treatment with different concentrations of SRT1720 promotes the proliferation of HUVECs at different times, and treatment with EX527 causes the opposite effect. All data are presented as the mean ± SD, *n* = 3. *p < 0.05 **p < 0.01 ***p < 0.001 relative to the control group. Differences were analysed using one‐way ANOVA. ANOVA, analysis of variance; HUVEC, human umbilical vein endothelial cells; qRT‐PCR, quantitative real‐time polymerase chain reaction; SD, standard deviation; SIRT1, sirtuin 1.

**FIGURE S2.** Different cell ratios and exposure times were determined for the angiogenic and osteogenic capacities of co‐culture system. (A) The spontaneous formation of vascular network in the co‐culture system at at different time points. (B) ALP staining of the osteoblast at different time points. The red box means the cell ratio and time chosen for further angiogenic and osteogenic experiments.

**FIGURE S3.** SIRT1 activation makes no effect on the osteogenic capacities of osteoblast. (A) ALP and ARS staining of the osteoblast at different time points. (B) qRT‐PCR analysis of osteogenic genes in the osteoblast at 7 days. (C) Western blot analysis of osteogenic proteins in the osteoblast at 7 days. Scale bar = 500 μm. All data are presented as the mean ± SD, *n* = 3. *p < 0.05 **p < 0.01 ***p < 0.001 relative to the control group. Differences were analysed using one‐way ANOVA. ALP, alkaline phosphatase; ANOVA, analysis of variance; ARS, alizarin red; O, osteoblast; qRT‐PCR, quantitative real‐time polymerase chain reaction; SD, standard deviation; SIRT1, sirtuin 1.

**FIGURE S4.** Quantitative analysis of western blot bands in Figure 1. *n* = 3. *p < 0.05 **p < 0.01 ***p < 0.001 relative to the control group. Differences were analysed using one‐way ANOVA.

**FIGURE S5.** Quantitative analysis of western blot bands in Figure 2. *n* = 3. *p < 0.05 **p < 0.01 ***p < 0.001 relative to the control group. Differences were analysed using one‐way ANOVA.

**FIGURE S6.** Quantitative analysis of western blot bands in Figure 3. *n* = 3. *p < 0.05 **p < 0.01 ***p < 0.001 relative to the control group. Differences were analysed using one‐way ANOVA.

**TABLE S1.** Primer sequences used in this study.
**TABLE S2.** Antibodies used for western blot and immunofluorescence.
**TABLE S3.** Primer sequences of Si RNA used in this study.

## Data Availability

All data generated or analysed during this study are included in this published article and its supplementary information files. The data that support the findings of this study are available from the corresponding author upon reasonable request.
