# Supplementary figures and images for: SIRT1 activation promotes bone repair by enhancing the coupling of type H vessel formation and osteogenesis
Source: Cell Prolif. 2024 Jan 11;57(6):e13596. doi: 10.1111/cpr.13596 (PMC11150139; doi:10.1111/cpr.13596)

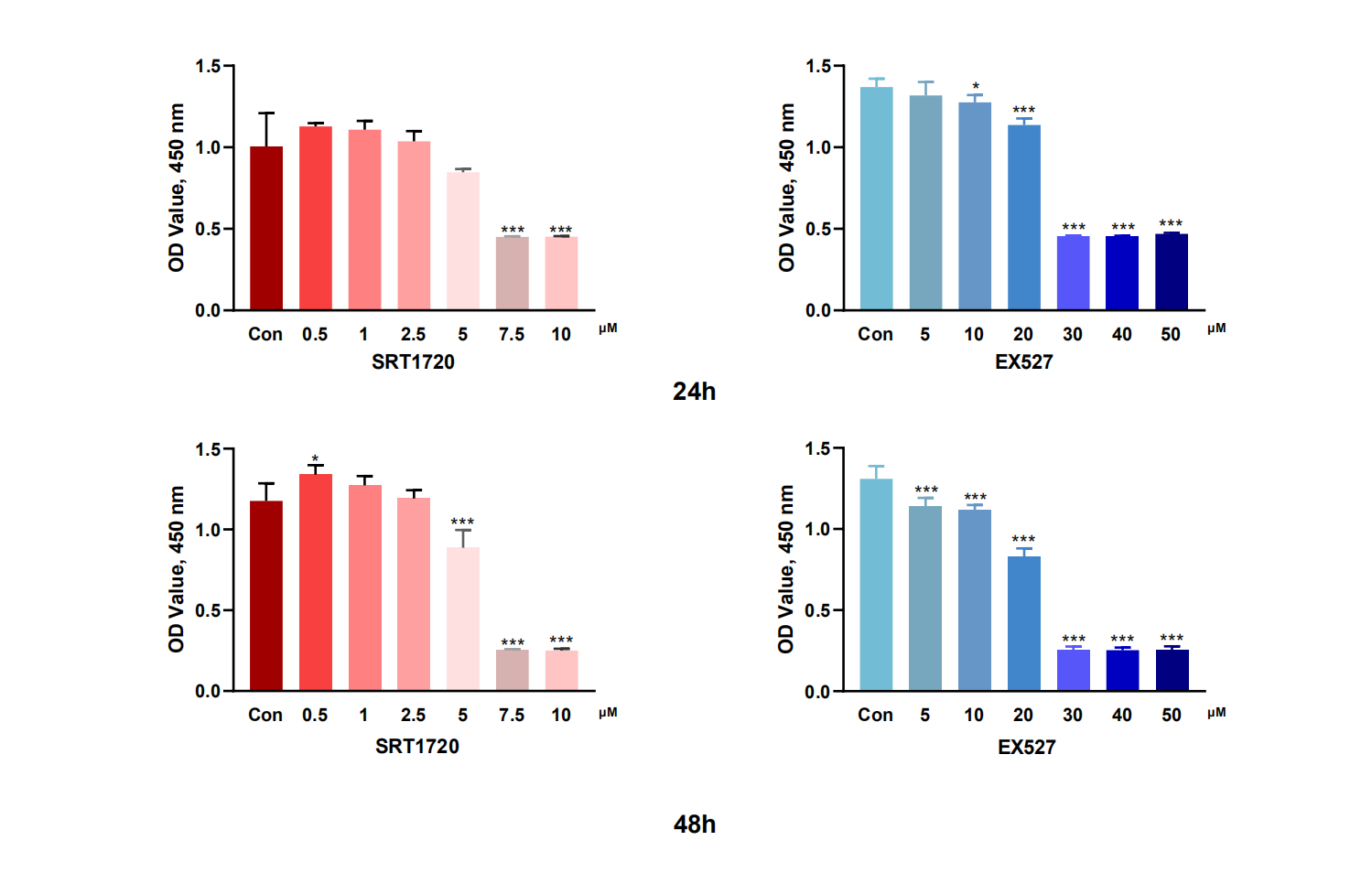

Supplement: Supplementary file 1 — FIGURE S1. SIRT1 activation promotes the proliferation of HUVECs. (A) Treatment with different concentrations of SRT1720 promotes the proliferation of HUVECs at different times, and treatment with EX527 causes the opposite effect. All data are presented as the mean ± SD, n = 3. *p < 0.05 **p < 0.01 ***p < 0.001 relative to the control group. Differences were analysed using one‐way ANOVA. ANOVA, analysis of variance; HUVEC, human umbilical vein endothelial cells; qRT‐PCR, quantitative real‐time polymerase chain reaction; SD, standard deviation; SIRT1, sirtuin 1. [file CPR-57-e13596-s005.tif]

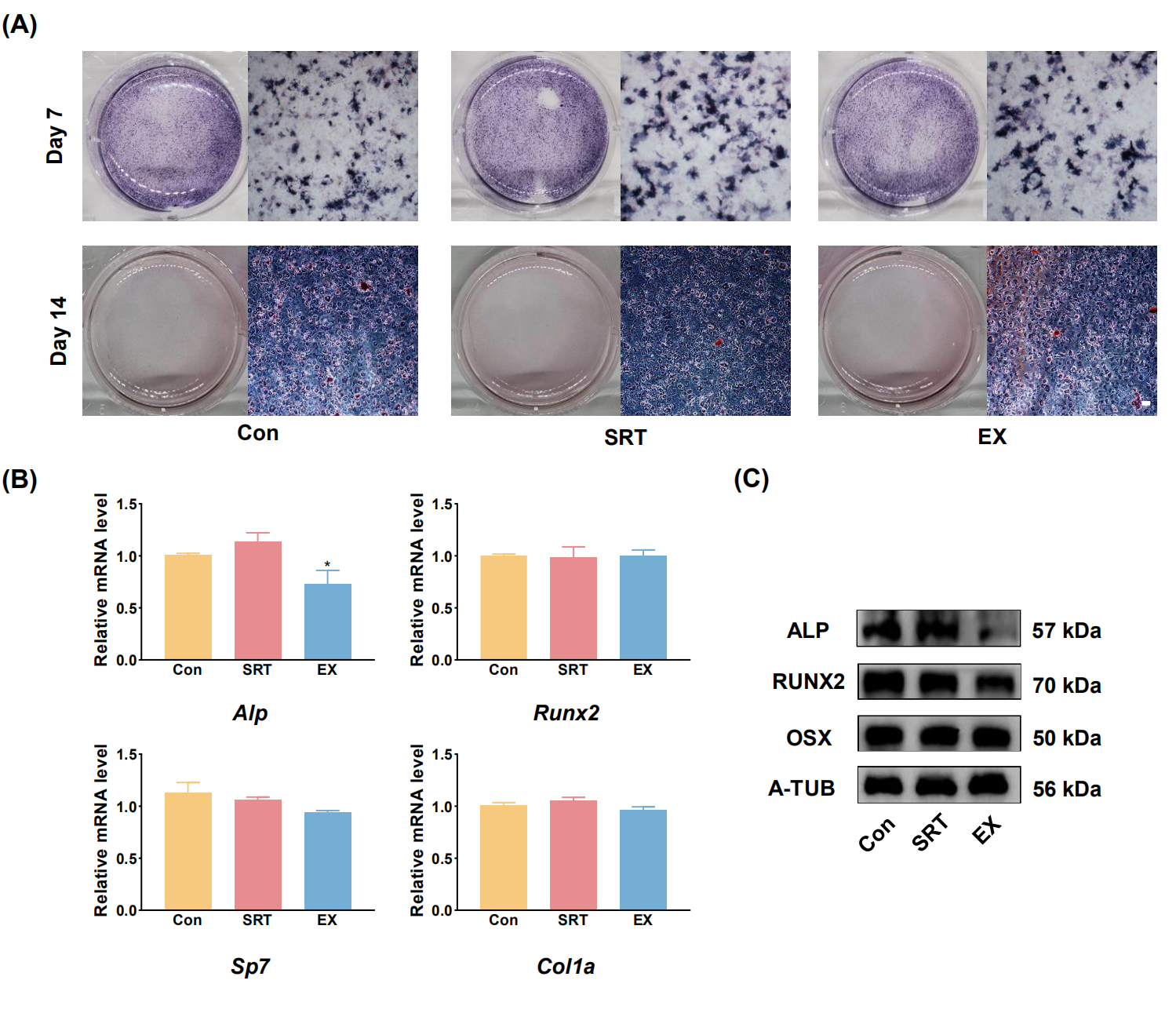

Supplement: Supplementary file 2 — FIGURE S2. Different cell ratios and exposure times were determined for the angiogenic and osteogenic capacities of co‐culture system. (A) The spontaneous formation of vascular network in the co‐culture system at at different time points. (B) ALP staining of the osteoblast at different time points. The red box means the cell ratio and time chosen for further angiogenic and osteogenic experiments. [file CPR-57-e13596-s001.tif]

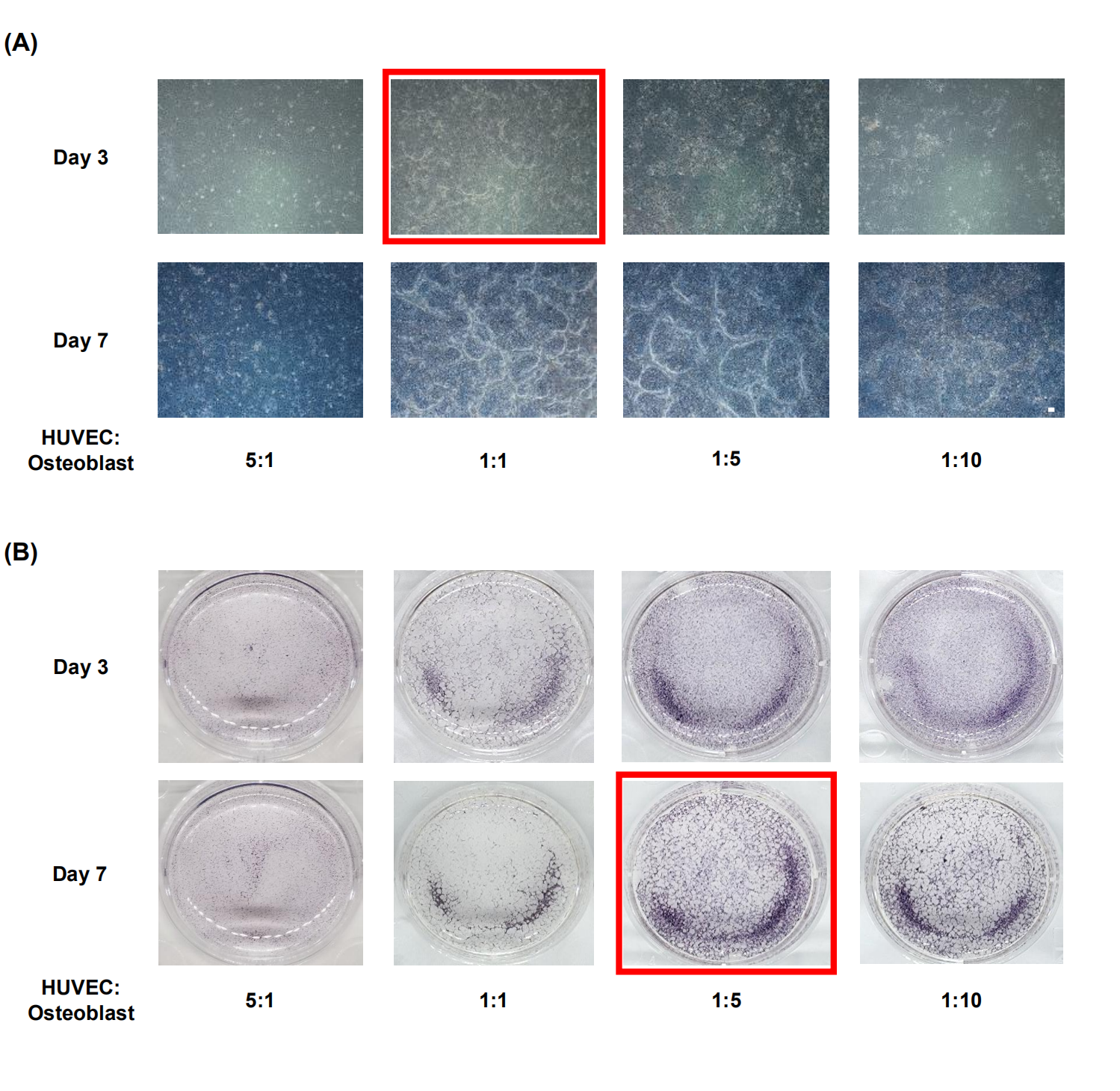

Supplement: Supplementary file 3 — FIGURE S3. SIRT1 activation makes no effect on the osteogenic capacities of osteoblast. (A) ALP and ARS staining of the osteoblast at different time points. (B) qRT‐PCR analysis of osteogenic genes in the osteoblast at 7 days. (C) Western blot analysis of osteogenic proteins in the osteoblast at 7 days. Scale bar = 500 μm. All data are presented as the mean ± SD, n = 3. *p < 0.05 **p < 0.01 ***p < 0.001 relative to the control group. Differences were analysed using one‐way ANOVA. ALP, alkaline phosphatase; ANOVA, analysis of variance; ARS, alizarin red; O, osteoblast; qRT‐PCR, quantitative real‐time polymerase chain reaction; SD, standard deviation; SIRT1, sirtuin 1. [file CPR-57-e13596-s003.tif]

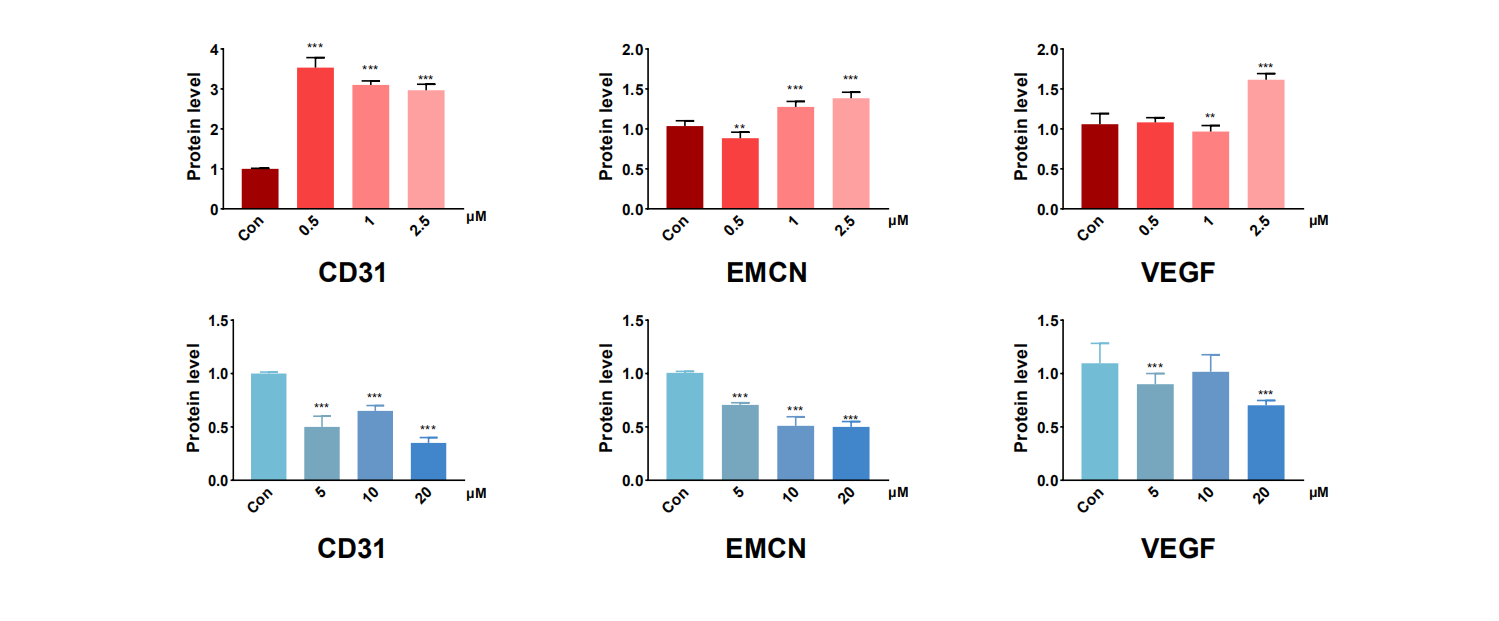

Supplement: Supplementary file 4 — FIGURE S4. Quantitative analysis of western blot bands in Figure 1. n = 3. *p < 0.05 **p < 0.01 ***p < 0.001 relative to the control group. Differences were analysed using one‐way ANOVA. [file CPR-57-e13596-s002.tif]

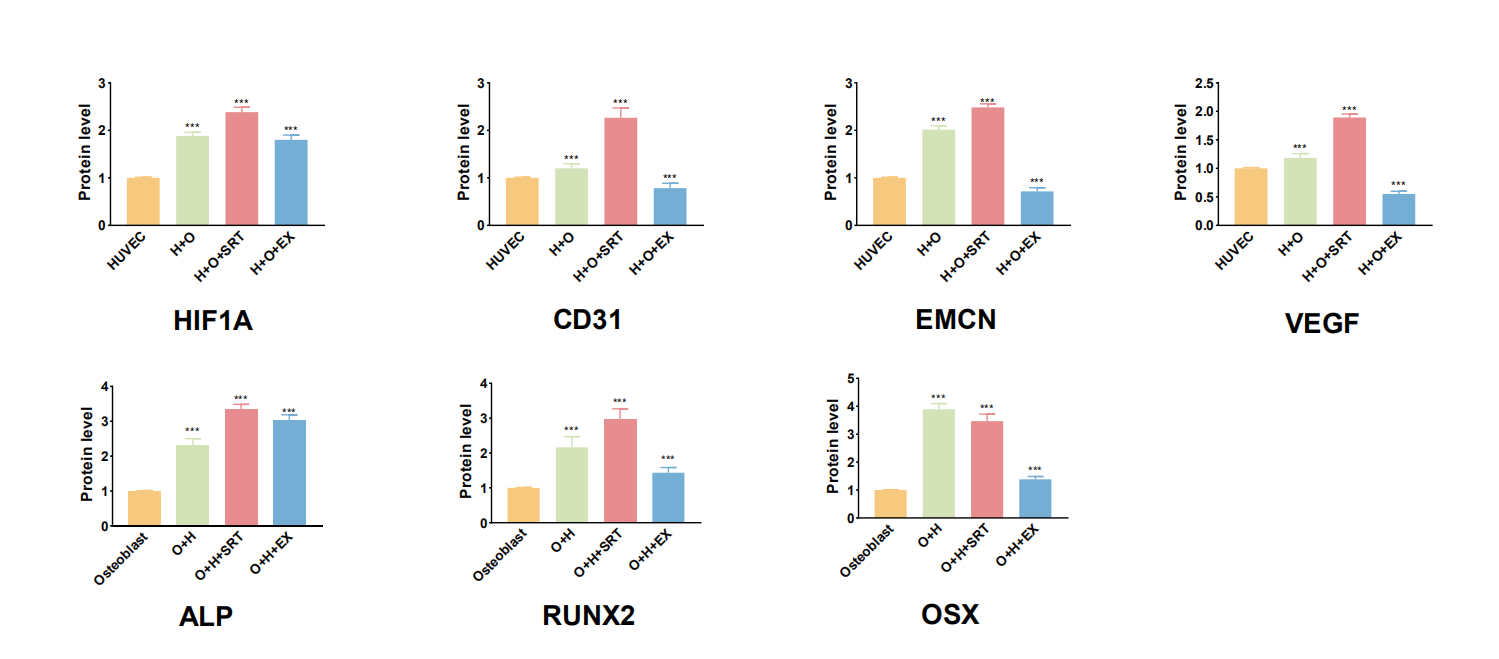

Supplement: Supplementary file 5 — FIGURE S5. Quantitative analysis of western blot bands in Figure 2. n = 3. *p < 0.05 **p < 0.01 ***p < 0.001 relative to the control group. Differences were analysed using one‐way ANOVA. [file CPR-57-e13596-s006.tif]

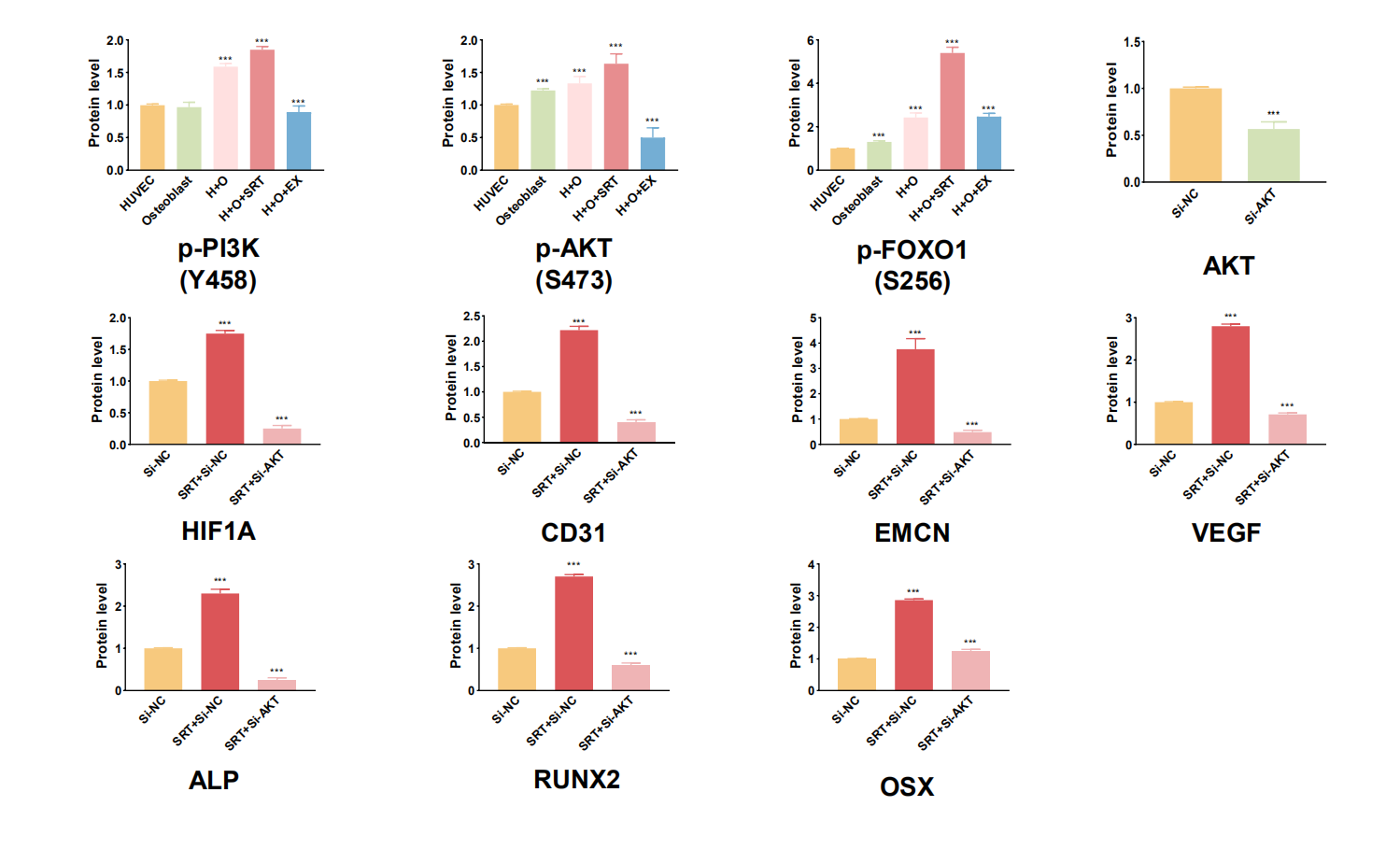

Supplement: Supplementary file 6 — FIGURE S6. Quantitative analysis of western blot bands in Figure 3. n = 3. *p < 0.05 **p < 0.01 ***p < 0.001 relative to the control group. Differences were analysed using one‐way ANOVA. [file CPR-57-e13596-s007.tif]
