# Supplementary material for: SIRT1 activation promotes bone repair by enhancing the coupling of type H vessel formation and osteogenesis
Source: Cell Prolif. 2024 Jan 11;57(6):e13596. doi: 10.1111/cpr.13596 (PMC11150139; doi:10.1111/cpr.13596)
Supplement: Supplementary file 7 — TABLE S1. Primer sequences used in this study. TABLE S2. Antibodies used for western blot and immunofluorescence. TABLE S3. Primer sequences of Si RNA used in this study. [file CPR-57-e13596-s004.docx]

**Table S1 Primer sequences used in this study**

| Gene | Forward Primer | Reverse Primer |
| --- | --- | --- |
| *GAPDH (human)* | GGAGCGAGATCCCTCCAAAAT | GGCTGTTGTCATACTTCTCATGG |
| *VEGF (human)* | AGGGCAGAATCATCACGAAGT | AGGGTCTCGATTGGATGGCA |
| *CD31 (human)* | GGTGGAGTCTGGAGAGGACATT | GGGTGGCATTTGAGGTCATT |
| *EMCN (human)* | AAGCCACAACCACTGATGTC | ACTCTGAGTTTCAGTCTTGGGT |
| *GAPDH (mouse)* | AGGTCGGTGTGAACGGATTTG | TGTAGACCATGTAGTTGAGGTCA |
| *ALP (mouse)* | CCAACTCTTTTGTGCCAGAGA | GGCTACATTGGTGTTGAGCTTTT |
| *RUNX2 (mouse)* | TTCAACGATCTGAGATTTGTGGG | GGATGAGGAATGCGCCCTA |
| *OSX (mouse)* | ATGGCGTCCTCTCTGCTTG | TGAAAGGTCAGCGTATGGCTT |
| *COL-1A (mouse)* | GGTGAGCCTGGTCAAACGG | ACTGTGTCCTTTCACGCCTTT |

**Table S2 Antibodies used for western blot and immunofluorescence.**

| Antibody | Company，Country |
| --- | --- |
| Anti-α-tubulin antibody | HUABIO，China |
| Anti-VEGF antibody | Abcam，USA |
| Anti-CD31 antibody | HUABIO，China |
| Anti-CD31 antibody | Abcam，USA |
| Anti-HIF-1α antibody | HUABIO，China |
| Anti-EMCN antibody | HUABIO，China |
| Anti-EMCN antibody | Santa Cruz，USA |
| Anti-ALP antibody | HUABIO，China |
| Anti-RUNX2 antibody | HUABIO，China |
| Anti-RUNX2 antibody | Santa Cruz，USA |
| Anti-OSX antibody | Cell signaling，USA |
| Anti-p-PI3K（Y458）antibody | Cell signaling，USA |
| Anti-PI3K antibody | Cell signaling，USA |
| Anti-p-AKT（S473） antibody | Cell signaling，USA |
| Anti-AKT antibody | Cell signaling，USA |
| Anti-p-FOXO1（S256） antibody | Immunoway，USA |
| Anti-FOXO1 antibody | Immunoway，USA |
| HRP Conjugated Goat anti Rabbit IgG Goat Polyclonal Antibody | HUABIO，China |
| HRP Conjugated Goat anti Mouse IgG Goat Polyclonal Antibody | HUABIO，China |
| FITC Conjugated Goat anti-Rabbit IgG Goat Polyclonal Antibody | HUABIO，China |
| Dylight 549，Goat Anti-Rat IgG | Abbkine，USA |
| iFluor™ 647 Conjugated Goat anti-mouse IgG Goat Polyclonal Antibody | HUABIO，China |

**Table S3 Primer sequences of Si RNA used in this study**

| Si RNA | Forward | Reverse |
| --- | --- | --- |
| *Si-NC* | UUCUCCGAACGUGUCACGUTT | ACGUGACACGUUCGGAGAATT |
| *Si AKT (human)* | CUCCUCAAGAAUGAUGGCATT | UGCCAUCAUUCUUGAGGAGTT |
